# Radiosynthesis and Biological Evaluation of *N*-[^18^F]Labeled Glutamic Acid as a Tumor Metabolic Imaging Tracer

**DOI:** 10.1371/journal.pone.0093262

**Published:** 2014-03-28

**Authors:** Kongzhen Hu, Kan Du, Ganghua Tang, Shaobo Yao, Hongliang Wang, Xiang Liang, Baoguo Yao, Tingting Huang, Linquan Zang

**Affiliations:** 1 PET-CT Center, Department of Nuclear Medicine, The First Affiliated Hospital, Sun Yat-Sen University, Guangzhou, China; 2 School of Pharmacy, Guangdong Pharmaceutical University, Guangzhou, China; Carl-Gustav Carus Technical University-Dresden, Germany

## Abstract

We have previously reported that *N*-(2-[^18^F]fluoropropionyl)-L-methionine ([^18^F]FPMET) selectively accumulates in tumors. However, due to the poor pharmacokinetics of [^18^F]FPMET *in vivo*, the potential clinical translation of this observation is hampered. In this study, we rationally designed and synthesized [^18^F] or [^11^C]labeled *N*-position L-glutamic acid analogues for tumor imaging. *N*-(2-[^18^F]fluoropropionyl)-L-glutamic acid ([^18^F]FPGLU) was synthesized with a 30±10% (n = 10, decay-corrected) overall radiochemical yield and a specific activity of 40±25 GBq/μmol (n = 10) after 130 min of radiosynthesis. *In vitro* cell experiments showed that [^18^F]FPGLU was primarily transported through the X_AG_^–^ system and was not incorporated into protein. [^18^F]FPGLU was stable in urine, tumor tissues, and blood. We were able to use [^18^F]FPGLU in PET imaging and obtained high tumor to background ratios when visualizing tumors tissues in animal models.

## Introduction

Positron emission tomography (PET) provides noninvasive images of physiologic function and is now regularly used in the diagnosis and staging of cancer [1]. Targeting of tumor specific metabolic pathways represents a useful strategy for early tumor detection and characterization. At present, a glucose analog, [^18^F]fluoro-2-deoxy-D-glucose ([^18^F]FDG) as a widely used PET tumor tracer, is based on increased glucose utilization in tumor cells [2]. However, [^18^F]FDG has several disadvantages and limitations. [^18^F]FDG has a high uptake into the brain and nonmalignant, inflammatory cellular elements [3]. In addition, a noticeable portion of tumors with a low metabolic rate, such as prostate [4], [5], bladder, renal cancer, and other [^18^F]FDG non-avid cancers [6], are [^18^F]FDG PET-negative and cannot be detected by [^18^F]FDG PET. To be able to overcome these disadvantages, work has been conducted to identify new tracers for tumor imaging that has greater sensitivity and better specificity for tumor imaging. Since tumor growth and proliferation require a high amount of energy and specific amino acids, positron-emitting radionuclide-labeled amino acids can be potential alternatives for tumor imaging. For example, *S*-[^11^C]methyl-L-methionine ([^11^C]MET) and *O*-2-[^18^F]fluoroethyl-L-tyrosine ([^18^F]FET) are commonly used amino acid tracer for the *in vivo* targeting of brain tumors [7]. Furthermore, [^18^F]FET might be able to distinguish between tumor and inflammatory tissues. Unfortunately, one of the major limitations of extracranial tumor imaging with [^11^C]MET or [^18^F]FET has relative poor tumor uptake, producing low to moderate tumor to background ratios [8].

**Figure 1:**
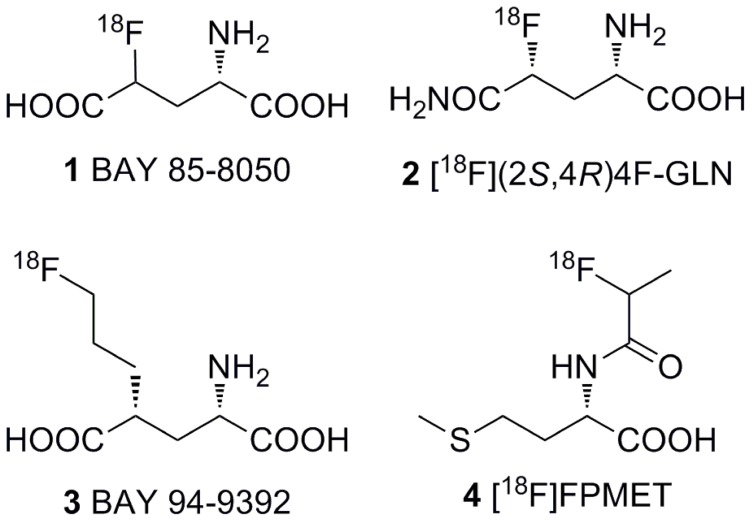
Structure of 1 (BAY 85-8050), 2 ([^18^F](2*S*, 4*R*)4F-GLN), 3 (BAY 94-9392), and 4 ([^18^F]FPMET).

Tumor cells use glucose and glutamine as their main sources of energy for both growth and proliferation. It has been reported that [^18^F]FDG-negative tumors may use a different metabolic pathway called–glutaminolysis [9]–[12]. Hence, in the adapted intermediary metabolism of tumors, it is possible that key roles are played by glutamine and glutamate. Recently, some [^18^F]labeled glutamate and glutamine derivatives have been reported [2], [13]–[16]. 4-[^18^F]fluoroglutamic acid (BAY 85-8050, **1**; see Figure 1) [13] and [^18^F](2*S*, 4*R*)-4-fluoroglutamine ([^18^F](2*S*, 4*R*)4F-GLN, **2**; see Figure 1) [2], [14] have demonstrated high uptake in tumor cells that undergo high growth and proliferation, but both of them become defluorinated in animals models, resulting in suboptimal images. Another derivative of glutamic acid, (4*S*)-4-(3-[^18^F]fluoropropyl)-L-glutamate (BAY 94-9392, **3**; see Figure 1) can be useful for detecting system X_C_^–^ (*SLC7A*11) activity *in vivo*, which is a potential biomarker for tumor oxidative stress. Animal models and use in humans have confirmed these observations [15], [16].

Our previous studies proposed the hypothesis that radiolabeled *N*-position amino acid analogues could function as potential PET tracers, which was confirmed by our early studies of *N*-(2-[^18^F]fluoropropionyl)-L-methionine ([^18^F]FPMET, **4,** see Figure 1) as a typical tool for tumor imaging [17]. Although intriguing uptake tumor was seen, its potential use in a clinical setting was hampered by poor stability and a high background signal *in vivo*. In the present study, we design and radiosynthesize *N*-(2-[^18^F]fluoropropionyl)-L-glutamic acid ([^18^F]FPGLU) and *N*-([^11^C]methyl)-L-glutamic acid ([^11^C]MGLU). Furthermore, we examine the biological evaluation of [^18^F]FPGLU as a stable PET tracer for tumor imaging.

## Materials and Methods

### General

All chemicals obtained commercially were used without further purification unless otherwise indicated. [^18^F]FDG was prepared as previously reported [18]. Sep-Pak light QMA, Plus C18, and Oasis HLB cartridges were obtained from Waters Corporation (Milford, MA, USA). Sep-Pak light QMA cartridges were preconditioned with 5 mL NaHCO_3_ aqueous (8.4%) and 10 mL water before use. Plus C18 and Oasis HLB cartridges were preconditioned with 10 mL ethanol and water before use. HPLC separation was carried out at the PET-MF-2V-IT-I synthesizer module (PET Co. Ltd., Beijing, China) built-in HPLC system with a semi-preparative reverse-phase C18 column (10×250 mm) equipped with a UV detector (Alltech 201, USA) and a radioactivity detector (PET Co. Ltd., China). The mobile phase was 55% solvent A (0.1% TFA in water): 45% solvent B (0.1% TFA in MeCN) with the flow rate of 4 mL/min. Analytical HPLC was performed using a Agilent 1200 Series HPLC system equipped with a ZORBAX Eclipse XDB-C18 analytic column (4.6×150 mm, 5 μm) using the flow rate of 1 mL/min. The gradient program started from 98% solvent A (0.1% TFA in water): 2% solvent B (0.1% TFA in MeCN) ramped to 90% solvent A: 10% solvent B at 8 min, and ramped to 20% solvent A: 80% solvent B at 20 min. The elution profile was detected with an ultraviolet detector (Agilent interface 35900E, Agilent Technologies, USA) at 210 nm and a B-FC-3200 high energy PMT Detector (Bioscan. Inc, Washington DC, USA). Radioactivity was measured by a calibrated ion chamber (Capintec CRC-15R) or a gamma counter (γ-counter) (GC-1200, USTC Chuangxin Co. Ltd. Zonkia Branch, China). No-carrier-added ^18^F-fluoride was obtained through the nuclear reaction ^18^O(p, n)^18^F by irradiation of more than 95% [^18^O]enriched water target with 10-MeV proton beam on the Cyclone 10/5 cyclotron (IBA Technologies, Belgium).

### Cell Culture and Animal Models

The S-180 Cell line and mice were obtained from the Laboratory Animal Center of Sun Yat-Sen University (Guangzhou, China). LTEP-a-2 and SPC-A-1 (SPCA-1) human lung adenocarcinoma cell lines were obtained from Shanghai Institute of Cellular Biology of Chinese Academy of Sciences (Shanghai, China). SPCA-1 cell line was originally isolated from the surgical specimens of a Chinese man with advanced lung adenocarcinoma by Shanghai Chest Hospital and Cellular Institute of Chinese Academy of Science in 1980 [19], [20]. The cells were cultured in RPMI 1640 medium with a physiologic glucose concentration (1.0 g/L) containing 5% fetal calf serum at 37°C in a humidified atmosphere of 5% CO_2_ and 95% air. Two days before the experiments in vitro, human lung adenocarcinoma SPCA-1 cell lines were trypsinized and 2×10^5^ cells per well were seeded into 12-well plates. Mice were housed 5 animals per cage under standard laboratory conditions at 25°C and 50% humidity. Tumor cells (1−2×10^7^) were injected subcutaneously and allowed to grow for 1 to 3 weeks. At the time of the experiments, the tumor reached 6−12 mm (diameter), the mice were 5–8 wk old, and weighed 18–28 g. All work studies were approved by the Institutional Animal Care and Utilization Committee (IACUU) of the First Affiliated Hospital, Sun Yat-Sen University (approval no.2012.001). All efforts were made to minimize animal suffering, to reduce the number of animals used and to utilize alternatives to in vivo techniques, if available.

### Synthesis of *N*-(2-fluoropropionyl)-L-glutamic Acid and *N-*methyl-L-glutamic Acid

The synthesis of *N*-(2-fluoropropionyl)-L-glutamic acid (FPGLU) was performed by a similar method as previously described [17]. Detail synthesis procedures for the FPGLU are included in File S1. The synthesis of *N-*methyl-L-glutamic acid (MGLU) was prepared based on previously procedures [21].

### Radiosynthesis of [^18^F]FPGLU

[^18^F]NFP (**8**) was prepared and dried based on previously procedures [22]. Anhydrous [^18^F]NFP was added a solution of diethyl L-glutamate hydrochloride (**9**, 100 μg) in DMSO (200 μL) and DIPEA (20 μL). The reaction mixture was heated for 5 min at 40°C and was quenched by adding of 5% acetic acid (600 μL) and was diluted with water (10 mL). The dilution was passed through a Plus C18 cartridge, and then the cartridge was washed with water (10 mL). The ester **10** was eluted with ether (5 mL) from the Plus C18 cartridge. The ether was evaporated under nitrogen flow at room temperature. To the residue was added NaOH aqueous (1 M, 50 μL). The ester **10** was hydrolyzed at room temperature for 8 min and the solution was neutralized with HCl (1 M, 50 μL). The product **11** was formulated in 0.9% saline and passed through a 0.22 μm Millipore filter for studies.

### Radiosynthesis of [^11^C]MGLU

^11^CO_2_ was produced by ^14^N(p, α)^11^C nuclear reactions using a Cyclone 10/5 cyclotron (IBA) and was delivered to the radiochemical laboratory. ^11^CO_2_ was trapped in a loop ring cooled with liquid nitrogen. ^11^CH_3_I was prepared from reduction of ^11^CO_2_ with LiAlH_4_, hydrolysis of the intermediately formed organmetallic complex, and subsequent iodination of ^11^C-methanol with hydrogen iodide. ^11^CH_3_I was translated to ^11^CH_3_OTf with AgOTf column [23]. ^11^CH_3_OTf was passed into a solution of diethyl L-glutamate hydrochloride (**9**, 2 mg) in acetone (1 mL) and DIPEA (40 μL). The reaction was reacted for 5 min and diluted with water (10 mL). The compound ester **12** was purified by plus C18 and eluted with ether (5 mL) from the Plus C18 cartridge. The ether was evaporated under nitrogen flow at room temperature. To the residue was added NaOH aqueous (1 M, 100 μL) and reacted at room temperature for 10 min. The solution was neutralized with HCl (1 M, 100 μL). The product **13** [^11^C]MGLU was formulated in 0.9% saline and passed through a 0.22 μm Millipore filter for studies.

### PET Studies

PET imaging of [^18^F]FPGLU (**11**), [^18^F]FPGLU ester (**10**), or [^11^C]MGLU (**13**) with S180 fibrosarcoma-bearing model mice (2 ∼ 3 mice per group) are included in File S1.

### Octanol-Water Partition Coefficient Study (logP)

Octanol-water partition coefficient for [^18^F]FPGLU was determined by measuring the distribution of radiolabeled compound in *n*-octanol and phosphate-buffered saline (pH = 7.4). A 20 μL sample of [^18^F]FPGLU (740 KBq, 20 μCi) in saline was added to a vial containing 5 mL each of *n*-octanol and phosphate-buffered saline. After being stirred in a vortex mixer for 1 min, the vial was centrifuged for 4 min to ensure complete separation of layers. Three hundred milliliters of each layer were measured using a γ-counter. logP value was calculated using the following formula: log_10_ P = log_10_ (counts in 0.3 mL of octanol/counts in 0.3 mL of water) [24].

### Competitive Inhibition Studies and Protein Incorporation

The methods of competitive inhibition and protein incorporation were similar as reported previously [17], [25], [26]. Detailed procedures for studying [^18^F]FPGLU using human lung adenocarcinoma SPCA-1 cells are included in File S1.

### *In Vivo* Biodistribution of [^18^F]FPGLU

For single-isotope (^18^F) biodistribution studies, Kunming mice (body weight range, 18–22 g) were anesthetized with 5% chloral hydrate solution (6 mL/kg) before injection of radiotracer. They were injected with 0.74−1.48 MBq (20−40 μCi) of [^18^F]FPGLU in 100−200 μL of saline through the tail vein. Radioactivity in the syringe before and after administration was measured in a calibrated ion chamber. The animals were killed by cervical dislocation at various times after injection, blood was obtained through the eyeball, and the organs of interest (blood, brain, heart, lung, liver, kidneys, pancreas, stomach, and intestine) were rapidly dissected and weighed, and ^18^F radioactivity was counted with a γ-counter. All measurements were background-subtracted and decay-corrected to the time of injection, then averaged together.

### Small-Animal PET Imaging

Small-Animal PET-CT imaging studies with tumor-bearing mice were carried out using the Inveon small-animal PET/computed tomography (CT) scanner (Siemens). 3.7−7.4 MBq of [^18^F]FPGLU were injected intravenously in conscious animals via the tail vein. Ten minutes later, the mice were anesthetized with 5% chloral hydrate solution (6 mL/kg) and were then placed on a heating pad to maintain body temperature throughout the procedure. Animals were visually monitored for breathing and any other signs of distress throughout the entire imaging period. Ten-minute static PET images were acquired at four time points (30, 60, 90 and 120 min) postinjection. For a comparative study, mice were kept fasting for 4 h and were anesthetized with 5% chloral hydrate solution (6 mL/kg) and imaged with [^18^F]FDG (3.7 MBq) at 60 min after intravenous injection. Imaging started with a low-dose CT scan, immediately followed by a PET scan. The CT scan was used for attenuation correction and localization of the lesion site. The images were reconstructed by two-dimensional ordered-subsets expectation maximum (OSEM). For each small-animal PET scan, ROIs were drawn over the tumor and major organs on decay-corrected whole-body coronal images using Inevon Research Workplace 4.1 software. The radioactivity concentration (accumulation) within a tumor or an organ was obtained from mean pixel values within the multiple ROI volume, which were converted to MBq/mL by using a conversion factor. Assuming a tissue density of 1 g/mL, the ROIs were converted to MBq/g and then divided by the administered activity to obtain an imaging ROI-derived %ID/g.

### *In Vitro* and *in Vivo* Stability

*In vitro* experiment, a sample of [^18^F]FPGLU (0.74 MBq, 20 μL) dissolved in normal saline was added to 200 μL of mouse serum and incubated at 37°C [27]. An aliquot of the serum sample was filtered through a 0.22 μm Millipore filter and was injected into a radio-HPLC column to analyze the stability of [^18^F]FPGLU in mouse serum within 2 h. *In vivo* stability experiment, Kunming mice bearing S180 fibrosarcoma were anesthetized with 5% chloral hydrate solution (6 mL/kg) and were injected with a dose of approximately 30 MBq (810 μCi) of [^18^F]FPGLU in 200 μL of normal saline via the tail vein [8]. The mice were sacrificed at 0.5 h and 1 h after injection. The amount of unchanged tracer in tissue samples was determined by radio-HPLC. Tumors were separately homogenized in phosphate-buffered saline (PBS) and centrifugation (18000 *g*, 6 min). Supernatant was filtered through a 0.22 μm Millipore filter and analyzed by radio-HPLC. Blood was obtained by extirpating eyeballs, centrifuging (18000 *g*, 6 min). The plasma was filtered through a 0.22 μm Millipore filter and analyzed by radio-HPLC. The urine was collected from the bladder and was injected into an HPLC column to analyze the metabolic fate of [^18^F]FPGLU in vivo.

### Statistical Analysis

Data were expressed as mean ± SD. Statistical analysis was performed with SPSS software, version 13.0 (SPSS Inc.), for Windows (Microsoft). Continuous variables were analyzed using the Student’s *t*-test. A P value of less than 0.05 was considered to indicate statistical significance.

## Results

### Radiochemistry

**Figure 2:**
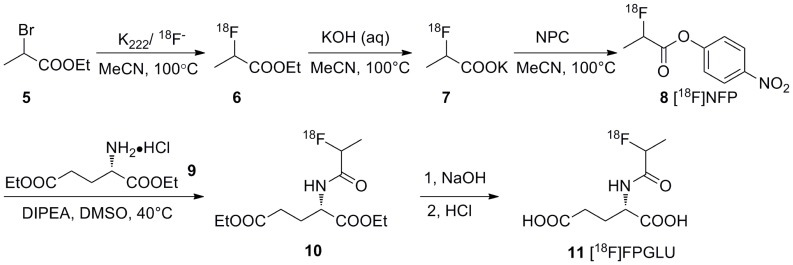
Scheme of the radiosynthesis of [^18^F]FPGLU.

**Figure 3:**
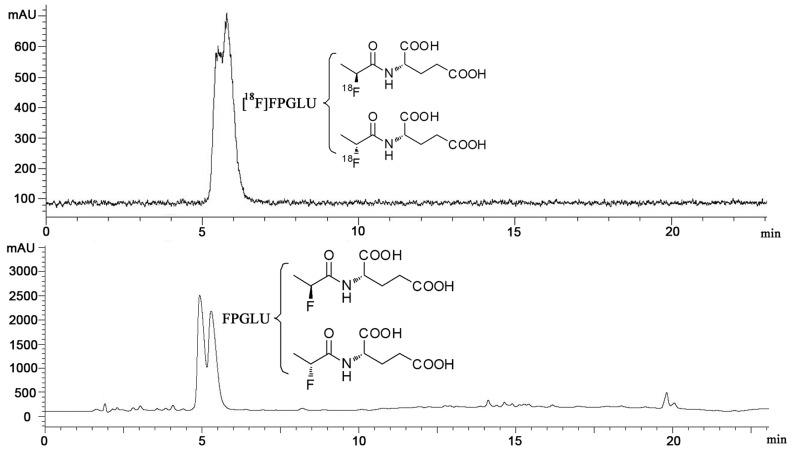
HPLC chromatograms of [^18^F]FPGLU and cold FPGLU. (Two peaks represent a mixture containing two epimers of [^18^F]FPGLU or FPGLU.).

Nonradioactive FPGLU and MGLU were synthesized for use as a reference standard for characterizing radioactive [^18^F]FPGLU and [^11^C]MGLU by HPLC. [^18^F]FPGLU **(11)** was synthesized as shown in Figure 2. The prosthetic group [^18^F]NFP **(8)** was synthesized in an automated process from ethyl 2-bromopropionate **(5)** as the precursor via a three-step, one-pot procedure on the modified PET-MF-2V-IT-I synthesizer [22]. Compound **9** was coupled with dried [^18^F]NFP and purified with a C18 cartridge. The compound **10** was further hydrolyzed with sodium hydroxide and neutralized with hydrochloric acid to afford **11** [^18^F]FPGLU. The decay-corrected radiochemistry yield of [^18^F]FPGLU was 90±5% (n = 10) from [^18^F]NFP for 30 min. The total decay-corrected radiochemical yield of [^18^F]FPGLU was 30±10% (n = 10) from ^18^F^−^ for 130 min, with a specific activity of 40±25 GBq/μmol (n = 10). As can be seen from Figure 3, [^18^F]FPGLU had a radiochemical purity of more than 95% and the tracer was initially evaluated as an epimeric mixture.

**Figure 4:**
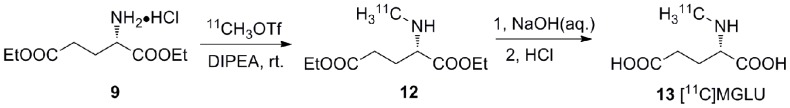
Scheme of the radiosynthesis of [^11^C]MGLU.

[^11^C]MGLU was synthesized as shown in Figure 4. Compound **9** was reacted with [^11^C]CH_3_OTf and purified with a C18 cartridge. The ester compound **12** was hydrolyzed and neutralized to afford the desire compound **13** [^11^C]MGLU. The decay-corrected radiochemistry yield of [^11^C]MGLU was 20±5% (n = 5) from [^11^C]CO_2_ for 60 min, with a specific activity of 100±30 GBq/μmol (n = 5).

### PET Studies

**Figure 5:**
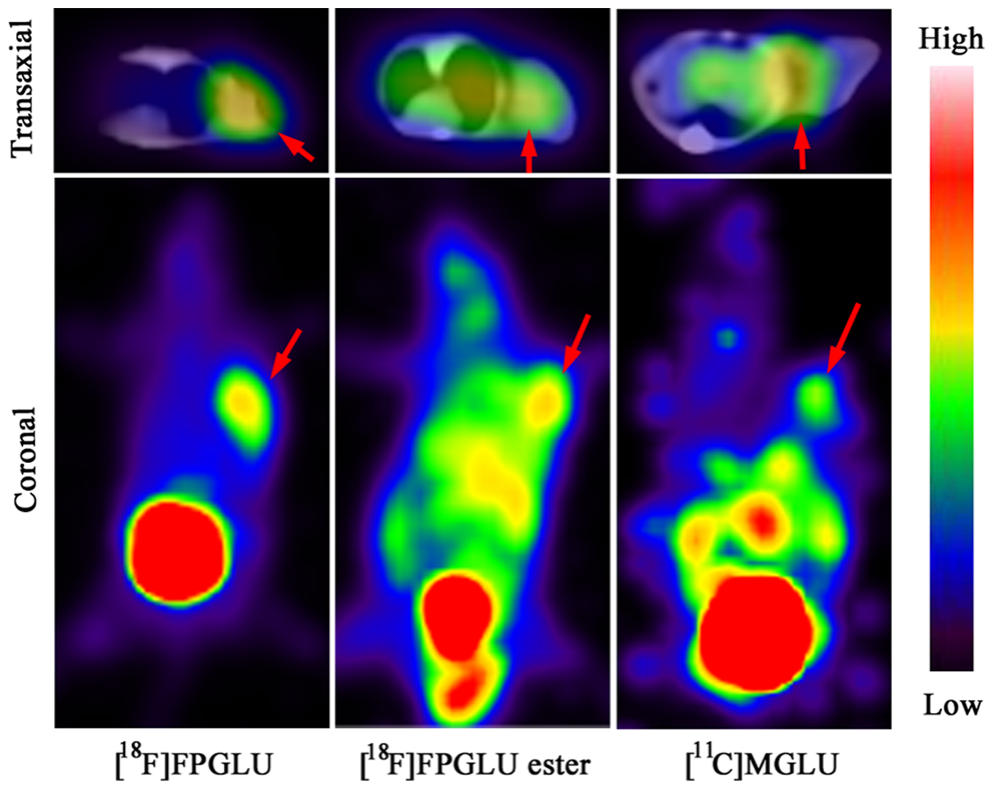
PET images of Kunming mouse with S180 fibrosarcoma. Images obtained with either [^18^F]FPGLU, [^18^F]FPGLU ester, or [^11^C]MGLU at 1 h after intravenous injection. Images are shown in both transaxial and coronal slices. (The red arrows indicate tumor.).

The whole-body [^18^F]FPGLU, [^18^F]FPGLU ester, and [^11^C]MGLU PET/CT imaging of S180 fibrosarcoma-bearing mice was performed. Comparative imaging studies demonstrated the superior performance of [^18^F]FPGLU over [^18^F]FPGLU ester or [^11^C]MGLU for imaging S180 fibrosarcoma, with excellent tumor-to-background contrast (Figure 5).

### Octanol-Water Partition Coefficient Study (logP)

The lipophilicity logP value of [^18^F]FPGLU at pH 7.4 is −1.54±0.06. As expected, the tracer was hydrophilic, as indicated by the logP of this derivative of an amino acid.

### Competitive Inhibition Studies

**Figure 6:**
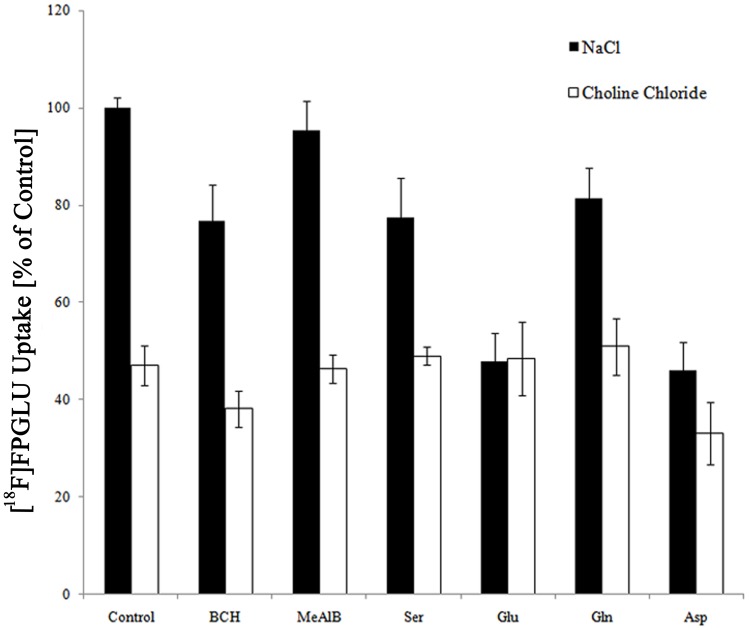
Uptake of [^18^F]FPGLU in SPCA-1 cells in presence of inhibitors for systems L, A, ASC, X_C_^–^, and X_AG_^–^ in the medium of presence and absence of Na^+^.

To investigate the transport mechanisms involved in the uptake of [^18^F]FPGLU, we conducted a series of competitive inhibition studies in SPCA-1 cells using specific inhibitors for system A, ASC, L, X_C_^–^, and X_AG_^–^, which are the major amino acid transport systems in mammalian cells and are potentially responsible for the uptake of glutamate and its analogs [14], [28], [29]. Figure 6 showed the results from these inhibition experiments when competing amounts of inhibitors were included and when Na^+^ was either present or absent. The data showed that the system A inhibitor *N*-methyl-2-amino-isobutyric acid (MeAIB) had no inhibitory effect on the uptake of [^18^F]FPGLU. In presence of Na^+^, the uptake of [^18^F]FPGLU was inhibited by 23% by the system L inhibitor 2-amino-2-norbornane-carboxylic acid (BCH). The system ASC inhibitors, L-serine (Ser) and L-glutamine (Gln), inhibited uptake of [^18^F]FPGLU by 23% and 19%, respectively. The system X_C_^–^ and X_AG_^–^ inhibitor L-glutamic acid (Glu) inhibited uptake of [^18^F]FPGLU by 52%, and the system X_AG_^–^ inhibitor D-aspartic acid (Asp) inhibited uptake of [^18^F]FPGLU by 54%. Replacement of NaCl by choline chloride reduced uptake of [^18^F]FPGLU by 53%. In the absence of Na^+^, BCH reduced uptake of [^18^F]FPGLU by 62%. Asp reduced uptake of [^18^F]FPGLU by 67%, and other inhibitors (MeAlB, serine, Gln, and Glu) did not markedly reduce the uptake of [^18^F]FPGLU. These results indicated that Na^+^-dependent X_AG_^–^, ASC, and Na^+^-independent L systems were involved in the transport of [^18^F]FPGLU, with X_AG_^–^ possibly playing a more dominant role.

### Protein Incorporation

SPCA-1 cells were incubated with [^18^F]FPGLU for 30 min, then trichloroacetic acid was used to precipitate them, which showed there was less than 1% of the radioactivity in the acid precipitable fraction. Therefore, there was no incorporation of [^18^F]FPGLU into the protein, which was similar to many labeled non-protein-composition amino acids tracers (e.g., BAY 85-8050, [^18^F]FPMET, 6-[^18^F]fluoro-L-dihydroxyphenylalanine, and *O*-(2-[^18^F]fluoroethyl)-L-tyrosine) [15], [17], [30], [31].

### *In Vivo* Biodistribution of [^18^F]FPGLU

The *in vivo* biodistribution data of [^18^F]FPGLU were summarized in Table 1. The rapid uptake of [^18^F]FPGLU was observed within the kidneys but it was quickly excreted through the urinary bladder, with 30.0 and 1.15%ID/g in the kidneys at 5 and 120 min, respectively. Blood clearance dropped fairly quickly with time, and showed low blood activity (1.04%ID/g) at 120 min after injection. [^18^F]FPGLU showed a moderate liver uptake with a relatively slow washout rate. Lung, spleen, intestine, heart, pancreas and stomach, showed relatively moderate uptake of radioactivity at 5 min, which decreased over the course of the 2 h study. There were negligible or very low levels (<1%ID/g) of uptake in other organs of interest, for example, the brain, bone, and muscle.

**Table 1:** Biodistribution of [^18^F]FPGLU in Normal Mice*^a.^*

Organ	5 min	30 min	60 min	120 min
Brain	0.68±0.21	0.83±0.10	0.89±0.21	0.75±0.08
Heart	1.80±0.50	1.68±0.07	1.27±0.32	0.98±0.25
Lung	3.06±0.55	2.00±0.43	1.17±0.27	0.92±0.07
Liver	3.53±0.12	3.27±0.80	2.07±0.56	1.35±0.17
Spleen	1.79±0.48	1.60±0.80	0.99±0.18	0.60±0.17
Pancreas	1.60±0.18	1.30±0.20	0.77±0.13	0.67±0.07
Kidneys	30.0±6.3	18.9±4.0	3.25±0.42	1.15±0.15
Intestine	1.41±0.29	1.56±0.19	1.03±0.23	0.96±0.04
Muscle	0.85±0.17	0.86±0.13	0.74±0.19	0.60±0.09
Stomach	1.51±0.46	1.78±0.62	0.94±0.13	0.87±0.19
Bone	0.92±0.05	0.85±0.09	0.75±0.20	0.53±0.06
Blood	2.17±0.48	1.91±0.21	1.54±0. 15	1.04±0.30

a Means ± SD (*n = *4). Data are average % ID/g.

### Small-Animal PET Imaging

**Figure 7:**
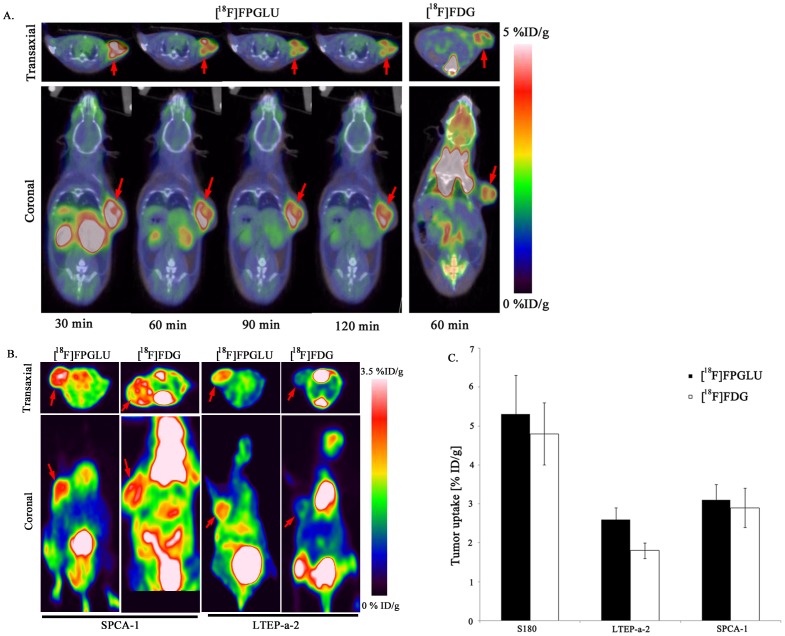
Small animal PET imaging and quantification. Decay-corrected whole-body PET images were acquired at different time points. (A) PET images of S180 fibrosarcoma-bearing mouse static scans at 0.5, 1, 1.5, and 2 h after the injection of [^18^F]FPGLU. The same S180 fibrosarcoma-bearing mouse static scan at 1 h after injection of [^18^F]FDG. (The red arrows indicate the tumor.) (B) PET images of SPCA-1 or LTEP-a-2 human lung adenocarcinoma-bearing nude mouse static scans at 1 h after the injection of [^18^F]FPGLU or [^18^F]FDG. (The red arrows indicate the tumor.) (C) **A** comparison of tumor uptake of [^18^F]FPGLU and [^18^F]FDG in S180 fibrosarcoma, LTEP-a-2, and SPCA-1 human lung adenocarcinoma at 1 h after injection. (n = 3 per group; bars represent means ± SD.).

Small-animal PET-CT imaging using [^18^F]FPGLU was performed on S180 fibrosarcoma, SPCA-1, and LTEP-a-2 human lung adenocarcinoma mouse models (n = 3 per group) (Figures 7A and 7B). The tumors were clearly visible with high contrast to the contralateral background (muscle, bone, brain, neck, and other tissues) within each animal model. PET imaging provided the consistent distribution data obtained from the ex vivo biodistribution result of [^18^F]FPGLU. During the early part of the experiment, prominent renal uptake was measured and accumulation in the bladder was observed, which suggests that the renal-bladder route was the main excretory system. As shown in Figures 7A and 7B, the tumor could be readily visualized with [^18^F]FPGLU. Regions of interest (ROIs) from the whole organ on the coronal images were measured so that the accumulation of the radioactivity in the small-animal PET scans could be quantified. The uptake between [^18^F]FPGLU and [^18^F]FDG in the S180 fibrosarcoma and SPCA-1 human lung adenocarcinoma has no significant difference at 1 h (5.3±1.0%ID/g vs. 4.8±0.8%ID/g for S180 fibrosarcoma, n = 3, P>0.05; 3.1±0.4%ID/g vs. 2.9±0.5%ID/g for SPCA-1 human lung adenocarcinoma, n = 3, P>0.05). In the LTEP-a-2 human lung adenocarcinoma, the uptake of [^18^F]FPGLU was significantly higher than that of [^18^F]FDG at 1 h (2.6±0.3%ID/g vs. 1.8±0.2%ID/g, n = 3, P<0.05) (Figure 7C).

### *In Vitro* and *in Vivo* Stability

**Figure 8:**
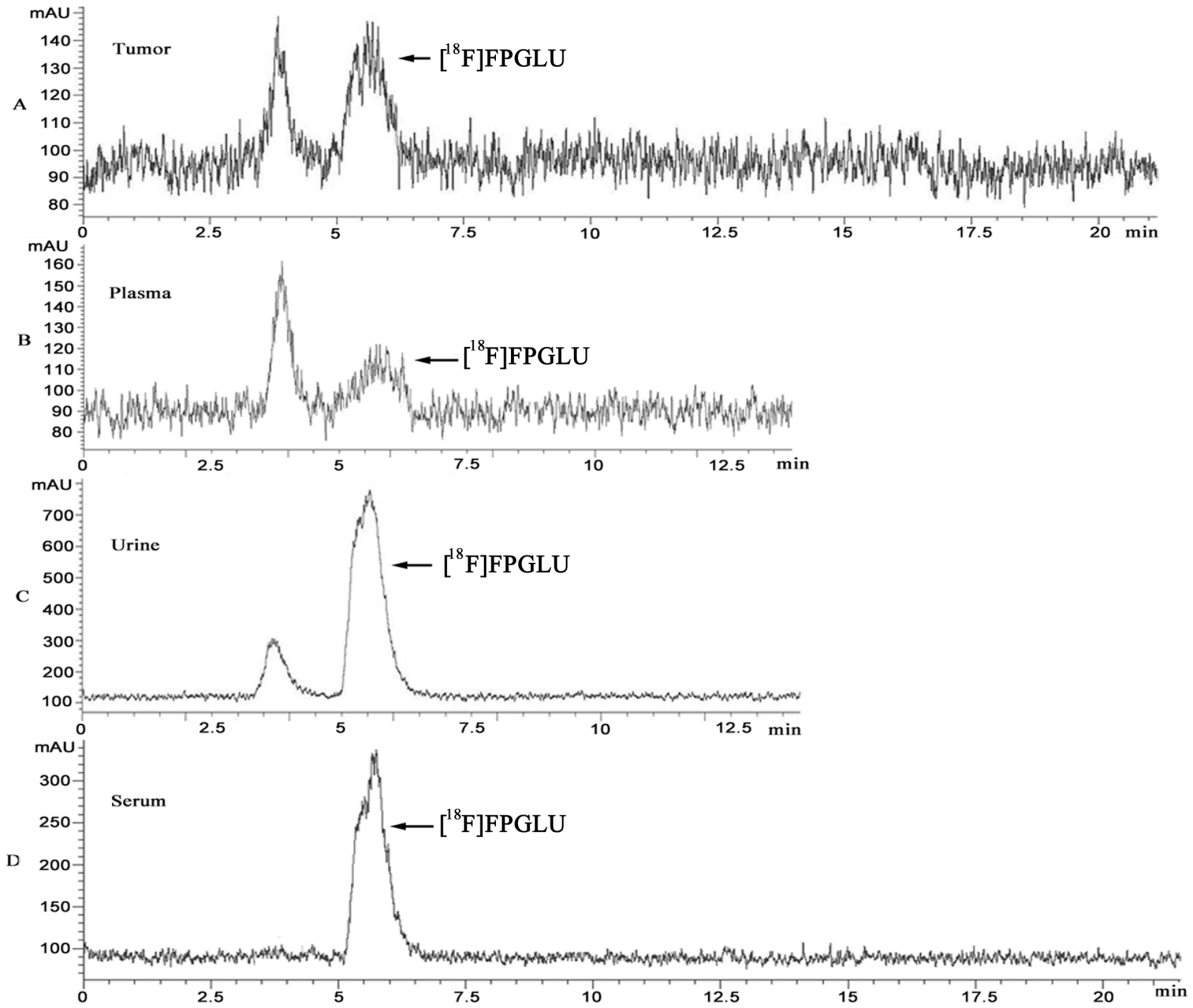
Radio-HPLC analysis the stability of [^18^F]FPGLU. HPLC chromatograms of tumor tissue extract (A) and plasma (B) (S180 fibrosarcoma-bearing mice at 0.5 h after intravenous injection of [^18^F]FPGLU). HPLC chromatograms of urine (C) (S180 fibrosarcoma-bearing mice at 1 h after intravenous injection of [^18^F]FPGLU). HPLC chromatograms of [^18^F]FPGLU in mouse serum at 37°C for 2 h (D). (The peak at T = 5.5 min was [^18^F]FPGLU.).

Radio-HPLC analysis showed that [^18^F]FPGLU in serum was stable. [^18^F]FPGLU in mouse serum at 37°C was more than 98% intact after 2 h. The stability of [^18^F]FPGLU *in vivo* and its metabolic fate was analyzed in urine, plasma, and tumor tissue (Figure 8). For the plasma and tumor tissue collected after 1 h, it was not possible to use HPLC to detect the radioactivity, as its level was too low. Therefore, data points for up to 0.5 h were the only ones that could be analyzed for the metabolites.

Radio-HPLC analysis of the extracted activity indicated that more than 40% of the [^18^F]FPGLU remained intact in the plasma 0.5 h post-injection. In tumor tissue, more than 65% of the tracer remained intact 0.5 h post-injection. The percentage of unchanged [^18^F]FPGLU in urine was 90% 0.5 h post-injection, which was reduced to 78% 1 h post-injection. HPLC analysis also showed that the main radioactive metabolites in tissues were highly polar products as they were eluted very fast.

## Discussion

Glutamate and its derivative play key roles in the adapted intermediary metabolism of tumors. Glutamate can be converted into and functions as a substitute for glutamine. Glutamate’s transamination product, α-ketoglutarate is directly channeled into the truncated tricarboxylic acid (TCA) cycle [13]. Its derivative *N*-acetyl-L-glutamate is the essential allosteric activator of the first urea cycle enzyme (carbamoyl phosphate synthetase I), which is a key regulator of this crucial cycle for ammonia detoxification in animals [32]. We have previously proposed that [^18^F]labeled *N*-acyl amino acids could be potential tracers for tumor imaging, as exemplified by [^18^F]FPMET. To further develop a more desirable PET tracer for tumor imaging with enhanced tumor-targeting efficacy and improved in vivo pharmacokinetics, we designed *N*-position positron-emitting radionuclide-labeled L-glutamic acid as novel tumor imaging tracers.

PET images of S180 fibrosarcoma-bearing mice with [^18^F]FPGLU, [^18^F]FPGLU ester, or [^11^C]MGLU showed radioactivity selectively accumulated in tumor. Comparative PET imaging characteristics of [^18^F]FPGLU with [^18^F]FPGLU ester or [^11^C]MGLU showed that [^18^F]FPGLU was superior over [^18^F]FPGLU ester or [^11^C]MGLU for imaging S180 fibrosarcoma, with excellent tumor-to-background contrast.

Malignant tumor cells accumulate amino acids and their close analogs because increased levels of their transporters are produced by the tumor, which also has a greater rate of protein synthesis. Adaptations of amino acid transport systems in cancer cells can offer opportunities to provide novel diagnostic and therapeutic targets. The BAY 94-9392 targeting system X_C_^–^ is considered to be a potential tracer for tumor imaging [15], [16]. In our experiments, [^18^F]FPGLU transport in SPCA-1 cells consisted of three processes, two saturable Na^+^-dependent activities and a non-saturable Na^+^-independent route. In the presence of Na^+^, [^18^F]FPGLU uptake was moderately inhibited by serine since the Na^+^-dependent system ASC contributed to [^18^F]FPGLU transport. In the presence and absence of Na^+^, [^18^F]FPGLU uptake was moderately inhibited by BCH since the Na^+^-independent system L also contributed to [^18^F]FPGLU transport. Glu and Asp peotently inhibited the uptake of [^18^F]FPGLU in the presence of Na^+^. However, when Na^+^ was absent, the uptake of [^18^F]FPGLU was not further reduced by Glu. The X_AG_^–^ system is a Na^+^-dependent transport system for anionic amino acids [33]. Therefore, [^18^F]FPGLU uptake was primarily transported through Na^+^-dependent X_AG_^–^. Since [^18^F]FPGLU was initially evaluated as an epimeric mixture, it was possible that the amino acid transport mechanism of this epimeric mixture was different from that of its single epimer, primarily due to its specificity for the various amino acid transport systems. A more detailed study on the biological transport properties of its single epimer *in vivo* and *in vitro* would require further study.

The biodistribution of [^18^F]FPGLU in mice showed that its radioactivity rapidly accumulated in kidneys and was quickly excreted through the urinary-bladder route. The radioactivity in other tissues was relatively low during the entire observation time. In comparison with [^18^F]FPMET [17], [^18^F]FPGLU had a lower uptake in the organs except in the kidneys at 30 and 60 min. It was suggested that [^18^F]FPGLU had a lower background to signal ratio than that of [^18^F]FPMET *in vivo*. The *in vivo* biodistribution results were confirmed by small-animal PET imaging. Small-animal PET studies using [^18^F]FPGLU exhibited the expected high uptake and retention in S180 fibrosarcoma, SPCA-1, and LTEP-a-2 human lung adenocarcinoma mice models. Future studies would be further observed the tumor uptake of [^18^F]FPGLU in other subcutaneous human tumor mouse models.

[^18^F]FPGLU was stable in mouse serum at 37°C for 2 h. However, the tracer was metabolized in vivo, which could be due to the lack of peptidases for peptide bond cleavage in the serum [34]. The level in the tumor tissue of the intact [^18^F]FPGLU was greater than that in the plasma 0.5 h post-injection. Perhaps [^18^F]FPGLU was rapidly accumulated in specific tumors through amino acid transporters and therefore maintained long-time stability in the tumor. It has been reported that the stability of [^18^F]FPMET was 25% of the unchanged [^18^F]FPMET in plasma and 8% of the unchanged [^18^F]FPMET in urine 0.25 h post-injection [17]. In these tissues studied, [^18^F]FPGLU was more stable than [^18^F]FPMET.

## Conclusion

In this study, we successfully designed and prepared the new tracer [^18^F] and [^11^C]labeled *N*-position L-glutamic acid analogues, and examined its potential application in imaging analysis of solid tumors *in vivo*. [^18^F]FPGLU showed good uptake and good tumor-to-background contrast in S180 fibrosarcoma, SPCA-1, and LTEP-a-2 human lung adenocarcinoma mouse models. [^18^F]FPGLU was primarily transported through the Na^+^-dependent X_AG_^–^ system. In addition, [^18^F]FPGLU showed relatively favorable pharmacokinetics *in vivo*. This work has shown that it is possible to use [^18^F]FPGLU as a tumor metabolic imaging tracer with PET.

## Supporting Information

File S1 The synthesis of nonradioactive *N*-(2-fluoropropionyl)-L-glutamate (FPGLU); the detail procedures of PET imaging of [^18^F]FPGLU, [^18^F]FPGLU ester, or [^11^C]MGLU with S180 fibrosarcoma-bearing model mice; the detail procedures of competitive inhibition and protein incorporation experiments; and the H&E staining of LTEP-a-2 and SPCA-1 human lung tumor.
(DOC)
