# Supplementary material for: Radiosynthesis and Biological Evaluation of N-[18F]Labeled Glutamic Acid as a Tumor Metabolic Imaging Tracer
Source: PLoS One. 2014 Mar 28;9(3):e93262. doi: 10.1371/journal.pone.0093262 (PMC3969356; doi:10.1371/journal.pone.0093262)
Supplement: File S1 — The synthesis of nonradioactive N-(2-fluoropropionyl)-L-glutamate (FPGLU); the detail procedures of PET imaging of [18F]FPGLU, [18F]FPGLU ester, or [11C]MGLU with S180 fibrosarcoma-bearing model mice; the detail procedures of competitive inhibition and protein incorporation experiments; and the H&E staining of LTEP-a-2 and SPCA-1 human lung tumor. (DOC) [file pone.0093262.s001.doc]

**SUPPLEMENTAL INFORMATION**

**Radiosynthesis and Biological Evaluation of *N*-[^18^F]Labeled Glutamic Acid as A Tumor Metabolic Imaging Tracer**

**Kongzhen Hu ^1^, Kan Du ^2^, Ganghua Tang ^1^,*, Shaobo Yao ^1^, Hongliang Wang ^1^, Xiang Liang ^1^, Baoguo Yao ^1^,** **Tingting Huang ^1^,****Linquan Zang ^2^**

**1** PET-CT Center, Department of Nuclear Medicine, The First Afﬁliated Hospital, Sun Yat-Sen University, Guangzhou, China, **2** School of Pharmacy, Guangdong Pharmaceutical University, Guangzhou, China

* Correspondence author: Ganghua Tang, E-mail: gtang0224@126.com

Tel: 86-20-87755766-8315; Fax: 86-20-87755766-8315

First author is currently a PhD Candidate; E-mail: [stonglass@163.com](mailto:stonglass@163.com)

Tel: 86-20-87755766-8317; Fax: 86-20-87755766-8317

**Materials and Methods**

All chemicals obtained commercially were used without further purification unless otherwise indicated. ^1^H-NMR spectra were recorded on bruker 400 or 600 spectrometer. Chemical shifts are expressed in parts per million relative to internal Me_4_Si (0.00 ppm). Coupling constants are given in Hz, and coupling patterns are abbreviated as follows: s, singlet; d, doublet; t, triplet; q, quadruplet; m, mutiplet; and dt, doublet of triplet. Mass spectrometry (MS) was obtained on a Quattro/LC mass spectrometer by electro-spray ionization. Elemental analyses were performed using a Vario RL analyzer.

**Synthesis of** ***N*-(2-fluoropropionyl)-L-glutamate (FPGLU)**

The synthesis of FPGLU was performed by a similar method as previously described.^1^ Briefly, to a solution of ethyl 2-fluoropropanoate (165 mg, 1.40 mmol) in MeCN (32 mL) was added KOH aqueous (6 mL, 0.2 M) at room temperature. After being stirred for 30 min at 100 °C, the reaction mixture was cooled to 40 °C and removed the solvent under reduced pressure. The residue was added another MeCN (20 mL) and evaporated to remove the residual water. Anhydrous MeCN (50 mL) and bis(4-nitrophenyl) carbonate (NPC, 626 mg, 2.06 mmol) were added to the residue at room temperature. After being stirred for 1 h at 100 °C, the reaction mixture was cooled to 0 °C and was quenched with AcOH aqueous (5%, 15 mL). The resulting mixture was extracted with CH_2_Cl_2_ (3 × 30 mL). The combined organic phases were washed with brine (5 mL), dried with anhydrous Na_2_SO_4_, filtered and concentrated under reduced pressure. The residue was added a solution of diethyl L-glutamate hydrochloride (240 mg, 1.0 mmol) in anhydrous MeCN (10 mL) and DIPEA (1.0 mL) at room temperature. After being stirred for 80 min at 50 °C, the reaction was cooled to room temperature and added saturated Na_2_CO_3_ (5 mL). The resulting mixture was extracted with CH_2_Cl_2_ (3 × 10 mL). The combined organic phases were washed with saturated Na_2_CO_3_ (5 mL) and brine (5 mL), dried with anhydrous Na_2_SO_4_, filtered and concentrated under reduced pressure. The residue was purified by flash chromatography on silica gel (eluent: EtOAc/heptane 1:2) to afford diethyl 2-(2-fluoropropanamido)pentanedioate as a colorless oil. The compound was an epimeric mixture. ^1^H NMR (600 MHz, CDCl_3_) δ 7.01 (s, 1H), 5.03–4.84 (m, 1H), 4.54 (dd, *J* = 13.4, 8.1 Hz, 1H), 4.25–3.99 (m, 4H), 2.42–2.24 (m, 2H), 2.18 (tdd, *J* = 11.6, 8.0, 4.4 Hz, 1H), 2.03–1.94 (m, 1H), 1.55–1.45 (m, 3H), 1.26–1.15 (m, 6H). MS (ESI) *m/z* (%) 278 (M + H^+^).

To a solution of diethyl 2-(2-fluoropropanamido)pentanedioate (60 mg, 0.22 mmol) in mixture solvent EtOH/H_2_O (10 mL, 4/1) was added NaOH aqueous (1 mL, 1.5 M) at 0 ºC. After being stirred for 1 h at 0 ºC, the pH of the reaction mixture was adjusted to 3~4 with HCl (2M) and the reaction mixture was extracted with CH_2_Cl_2_ (3 × 50 mL). The combined organic phases were dried with anhydrous Na_2_SO_4_, filtered and concentrated under reduced pressure. The residue was added MeCN and filtered, the solvent was removed from the filtrate to afford the FPGLU as a colorless oil. FPGLU was further purified by flash chromatography on silica gel (eluent: EtOAc/MeOH 1:4). FPGLU was an epimeric mixture. ^1^H NMR (400 MHz, MeOD) δ 5.09–4.92 (m, 1H), 4.46 (dd, *J* = 8.4, 4.4 Hz, 1H), 2.41 (m, 2H), 2.30–2.19 (m, 1H), 2.10–1.98 (m, 1H), 1.57 (dd, *J* = 6.8, 3.0 Hz, 1.5H), 1.51 (dd, *J* = 6.8, 3.0 Hz, 1.5H). MS (ESI) *m/z* (%) 222 (M + H^+^). Anal. Calcd for C_8_H_12_FNO_5_: C, 43.44; H, 5.47; N, 6.33. Foud: C, 43.30; H, 5.60; N, 6.57.

**PET Studies**

The S180 fibrosarcoma-bearing model mice were randomized into three groups. Each group (n = 2 ~ 3) was anesthetized with 5% chloral hydrate solution (6 mL/kg) before injection of the radiotracer and remained anesthetized throughout the study. PET Imaging was performed 60 min after administration of 0.93-1.86 MBq (25-50 μCi) of [^18^F]FPGLU, [^18^F]FPGLU ester, or [^11^C]MGLU via injection into the tail vein. The PET data were acquired at 60 min after intravenous injection in 3-dimensional mode, with emission scans of 10 min per bed position. Imaging started with a low-dose CT scan (30 mAs), immediately followed by a PET scan. The CT scan was used for attenuation correction and localization of the lesion site. The coronal, transaxial, and sagittal views of PET imaging and MIP (maximum intensity projection) of the model mice were obtained after image reconstruction with a slice thickness of two-micrometer.

**Competitive Inhibition Studies in Cells**

The inhibition experiments to characterize the amino acid transport pathway were used with the specific competitive inhibitors for system L: 2-amino-2-norbornane-carboxylic acid (BCH); for system A: *N*-methyl-2-amino-isobutyric acid (MeAIB); for system ASC: serine and L-glutamine (Gln); for system X_C_^–^: L-glutamic acid (Glu); and for system X_AG_^–^ inhibitor D-aspartic acid (Asp) and Glu. The concentration of the inhibitors used was 1 mmol/L. All experiments were performed in the presence and absence of Na^+^. In the preparation procedure of [^18^F]FPGLU, NaOH was replaced by KOH. In Na^+^-free experiments, NaCl was replaced by choline chloride. The RPMI 1640 medium was discarded, and the cells were washed thrice with NaCl (137 mmol/L, 1.0 mL) or choline chloride (137 mmol/L, 1.0 mL). The cells were added 200 μL of [^18^F]FPGLU (1.85 MBq/mL) and 200 μL of one of the inhibitors were incubated at 37 °C for 10 min. After stopping the tracer uptake with 1 mL of ice-cold phosphate-buffered saline, the cells were washed thrice with phosphate-buffered saline at 4 °C and dissolved in 1.5 mL of 0.1 M NaOH plus 2% Triton X (Sigma Chemical Co.) and the activity measured by a γ-counter. Each experiment was done in triplicate, averaged, and repeated 3 times on different days.

**Protein incorporation**

The RPMI 1640 medium was discarded, then the cells were incubated 400 μL of [^18^F]FPGLU (1.85 MBq/mL) at 37 °C for 30 min. At the end of incubation, the radioactive medium was removed. The cells were washed thrice with ice-cold phosphate-buffered saline (1.0 mL) and detached with 0.5 mL ethylenediaminetetraacetic acid (1%), transferred into a tube and 0.5 mL 20% trichloroacetic acid was added. After 30 min on ice, the samples were centrifuged at 18000 rpm for 10 min. The supernatant was removed and the pellet was washed thrice with phosphate-buffered saline. The radioactivity in both the supernatant and the pellet was measured by a γ-counter. Protein incorporation was calculated as the percentage of acid precipitable radioactivity.


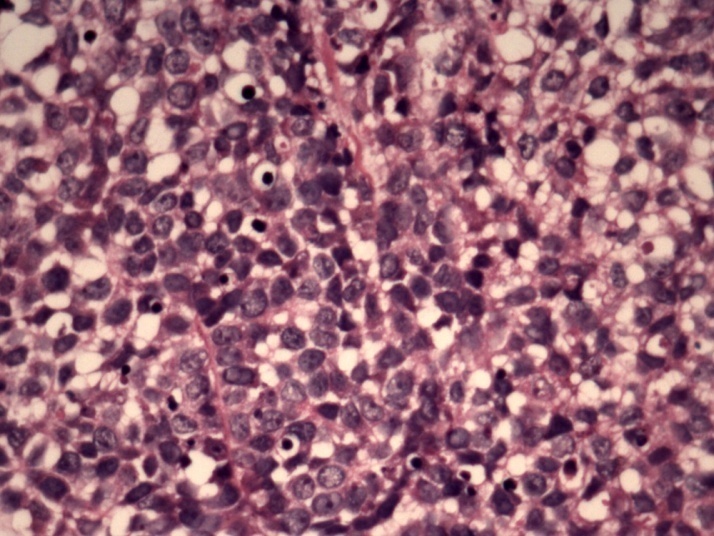


**A**


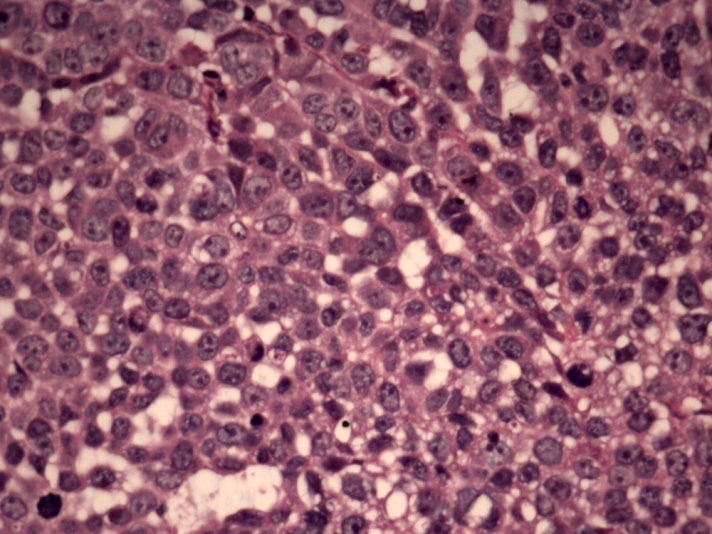


**B.A.**

**Figure S1 The pictures of H&E staining tumors.** LTEP-a-2 human lung tumor (A) and SPC-A-1 human lung tumor (B). Original magnification: ×40.

- **REFERENCES**

1. Hu KZ, Wang H, Huang T, [Tang G](http://www.ncbi.nlm.nih.gov/pubmed?term=Tang G%5BAuthor%5D&cauthor=true&cauthor_uid=23886847), [Liang X](http://www.ncbi.nlm.nih.gov/pubmed?term=Liang X%5BAuthor%5D&cauthor=true&cauthor_uid=23886847), et al. (2013) Synthesis and biological evaluation of *N*-(2-[^18^F]fluoropropionyl)-L-methionine for tumor imaging. [Nucl Med Biol](http://www.ncbi.nlm.nih.gov/pubmed/?term=Feasibility++of+Labeled++cx-Acetamido-Aminoisobutyric++Acid+as+New++Tracer+Compound++for++Kinetic-Labeling++of+Neutral++Amino++Acid+Transport%3A++Preparation++of+(x-+(N-%5B+1+J+‘C%5D+Ace) 40: 926–932.
